# Influenza A(H5N1) Virus Infections in 2 Free-Ranging Black Bears (*Ursus americanus*), Quebec, Canada

**DOI:** 10.3201/eid2910.230548

**Published:** 2023-10

**Authors:** Benjamin T. Jakobek, Yohannes Berhane, Marie-Soleil Nadeau, Carissa Embury-Hyatt, Oliver Lung, Wanhong Xu, Stéphane Lair

**Affiliations:** Centre Québécois sur la Santé des Animaux Sauvages/Canadian Wildlife Health Cooperative, St. Hyacinthe, Quebec, Canada (B.T. Jakobek, S. Lair);; Université de Montréal, St. Hyacinthe (B.T. Jakobek, S. Lair);; University of Saskatchewan Western College of Veterinary Medicine, Saskatoon, Saskatchewan, Canada (Y. Berhane);; Canadian Food Inspection Agency National Centre for Foreign Animal Disease, Winnipeg, Manitoba, Canada (Y. Berhane, C. Embury-Hyatt, O. Lung, W. Xu);; University of Manitoba, Winnipeg (Y. Berhane, O. Lung);; Ministère de l’Agriculture, des Pêcheries et de l’Alimentation Laboratoire de Santé Animale, St. Hyacinthe (M.-S. Nadeau)

**Keywords:** influenza, influenza A(H5N1) virus, influenza virus, viruses, infections, avian influenza, hemagglutinin, bears, free-ranging black bears, Ursus americanus, meningoencephalitis, zoonoses, Quebec, Canada

## Abstract

Wholly Eurasian highly pathogemic avian influenza H5N1 clade 2.3.4.4b virus was isolated from 2 free-ranging black bears with meningoencephalitis in Quebec, Canada. We found that isolates from both animals had the D701N mutation in the polymerase basic 2 gene, previously known to promote adaptation of H5N1 viruses to mammal hosts.

Since its arrival in North America during December 2021, the Eurasian highly pathogenic avian influenza (HPAI) virus subtype H5N1, clade 2.3.4.4b, has been associated with a high mortality rate for wild birds all over the continent (*1*). Affected bird species include mainly waterfowl and colonial nesting marine birds, as well as scavenger birds, such as gulls, eagles, vultures, and corvids (*2**,**3*). As with several other subtypes, HPAI H5N1 can potentially infect persons, although clinical cases in humans have been limited (*4*). However, this virus has been shown to be pathogenic for different species of wild mammals, including red fox, striped skunk, mink, raccoon, and seals (*5**–**7*). Infections with influenza A(H1N1) viruses have been described in captive sloth bears (*8*). We report and describe infections by HPAI H5N1 virus in 2 black bears (*Ursus americanus*) found in Quebec, Canada, during the summer of 2022.

## The Study

Two young-of-the-year black bear cubs (likely born in January‒February) were observed wandering on a road within the Forillon National Park in Gaspé, Quebec, Canada (48°51′39′′N, 64°13′26′′W), on June 14, 2022. The cubs, which were active and quite vocal, were not attended by their dam. Shortly afterward, an adult female bear with unusual behavior was reported ≈200 m from the cubs. This female was wandering between vehicles, fell into a river, and began circling. Upon the arrival of park officials, the animal was in lateral recumbency and convulsing in a ditch.

Because of the severity of the neurologic signs present and concern for public safety, the bear was anesthetized and then euthanized. The 2 cubs, presumed to be orphaned, were also euthanized. The carcasses of the adult female and 1 of the cubs were subsequently frozen. The adult female was thawed a few days later and examined on site. Different organs were sampled, refrozen and shipped, along with the originally frozen cub, to the Canadian Wildlife Health Cooperative Quebec regional center for further macroscopic examination, which was performed on July 7, 2022.

Macroscopic examination of viscera of the adult female show no notable findings, other than extensive postmortem changes. The cub (a 5.1-kg female) was thin and had limited fat stores. A mild mesenteric lymphadenomegaly was present, and the cerebrum appeared to be diffusely congested.

Histopathologic examination of tissues sampled from the adult female showed multifocal infiltration of the meninges by numerous lymphoplasmacytic cells, which extended into the Virchow–Robin spaces, forming perivascular cuffing. Neuronal necrosis associated with satellitosis and glial nodules was commonly observed. Aggregates of polymorphonuclear cells in the cerebral substance and areas of neuropile vacuolization with axonal degeneration were also present. The cub had similar, but of lower intensity and predominantly neutrophilic in nature, cerebral inflammatory and degenerative lesions, in addition to fibrinoid neutrophilic vasculitis. Small necrotic foci surrounded by granulocytes and mononuclear cells were also occasionally seen in the liver of the cub.

**Figure 1:**
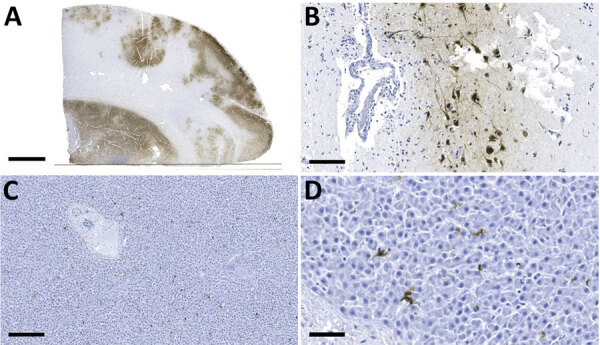
Detection of influenza A virus antigen in black bears by immunohistochemical analysis, Quebec, Canada. A) Brain tissue, showing abundant viral antigen detected multifocally throughout the section and observed primarily in gray matter areas. Scale bar indicates 5 mm. B) Brain immunostaining within neurons and surrounding neuropil. Scale bar indicates 100 μm. C) Liver tissue, showing viral antigen within individual cells. Scale bar indicates 200 μm. D) Liver tissue, showing that cells have the morphologic appearance of Kuppfer cells. Scale bar indicates 50 μm. Monoclonal antibody and diaminobenzidine stained, Gill’s hematoxylin counterstained.

Influenza A virus (IAV) antigen was detected by using immunohistochemical staining (Appendix) in brain and liver cells; liver cells were observed only in the bear cub (Figure 1). Brain tissues from both animals were negative for rabies virus by the direct rapid immunohistochemical test (*9*).

We extracted RNAs from brain tissues and tracheo-rectal swab specimens from both animals and found that they were positive for IAV genomic material by using matrix and H5 gene specific real-time reverse transcription PCRs (*10**,**11*). We isolated H5N1 viruses from brain samples collected from both bears in 9-day-old embryonated specific pathogen-free chicken eggs. We amplified all 8 genome segments of the virus directly from clinical samples and isolates and were sequenced by using the Oxford Nanopore platform as described (*12*), and the Rapid Barcoding Kit 96 (https://www.nanoporetech.com). We basecalled raw Nanopore signal data and demultiplexed with Guppy v5.1.12 (https://timkahlke.github.io) using the super-accurate basecalling model. We then analyzed and assembled the basecalled reads by using the CFIA-NCFAD/nf-flu v3.1.0 (https://zenodo.org/record/7011213#.ZBs5cXbMIuU) nextflow workflow.

**Figure 2:**
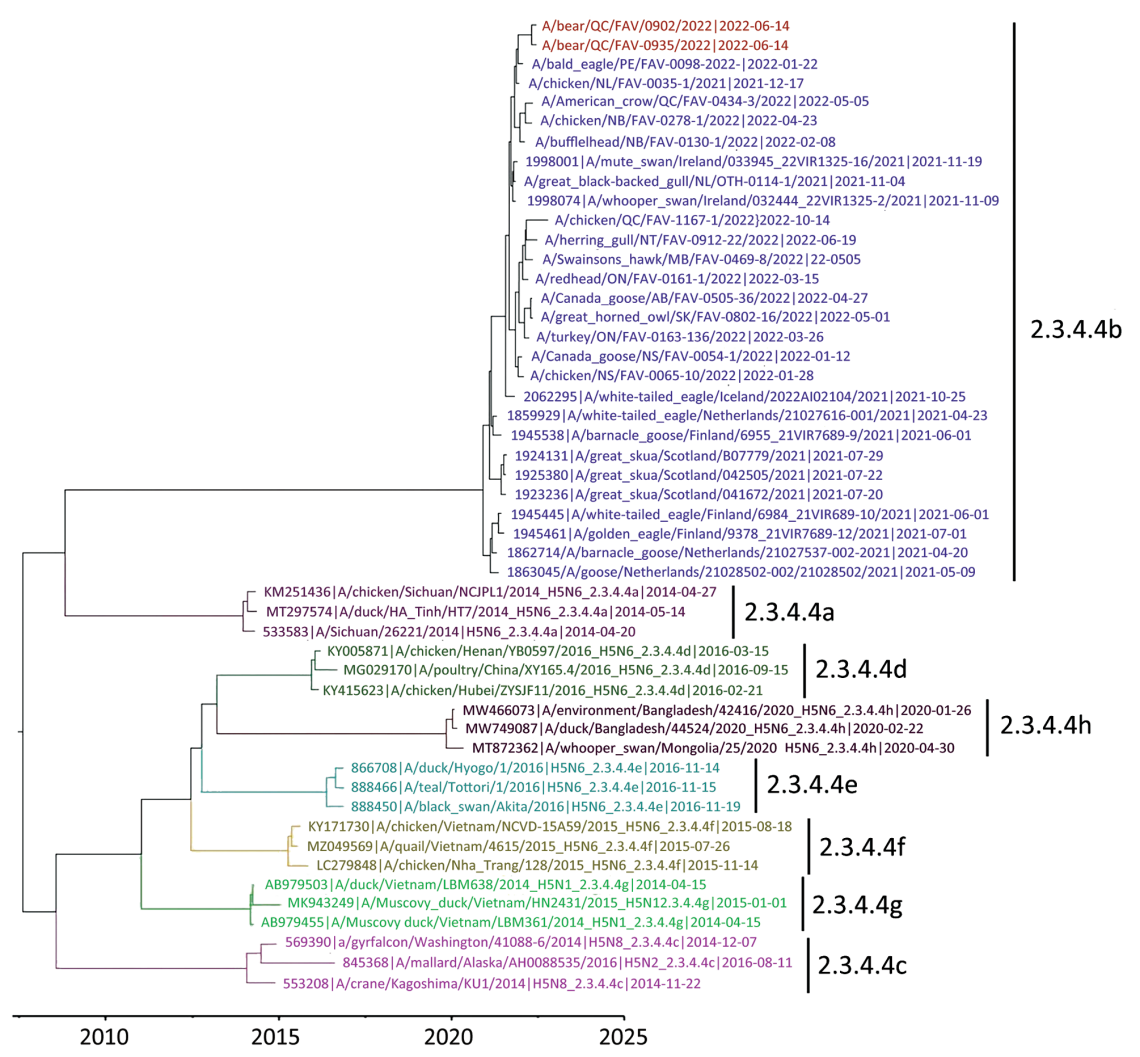
Maximum-clade credibility tree for influenza A virus antigen in black bears by immunohistochemical analysis, Quebec, Canada, inferred by using Bayesian and Markov Chain Monte Carlo analyses for the H5 hemagglutinin gene. Shown are relationships among black bear strains from this investigation (red), European 2021 H5 clade 2.3.4.4b HPAI strains (blue), and early Canada wild bird and poultry strains (purple). Colors and labels indicate the other H5 clade 2.3.4.4 subgroups.

 The hemagglutinin (HA) gene of the virus belonged to Eurasian goose/Guangdong (Gs/GD) lineage HPAI H5N1 clade 2.3.4.4b and had the cleavage site motif of PLREKRRKR/GLF, compatible with HPAI viruses (Figure 2). All 8 genome segments of the viruses from both bears contained wholly Eurasian IAVs similar to the Newfoundland-like H5N1 viruses, which came from Europe to Canada by the Atlantic Flyway in 2021 (*1*). Both bears had mammalian adaptive mutations (D701N) in the polymerase basic protein 2 subunit of the RNA polymerase complex.

We conducted a Bayesian phylogenetic analysis to identify phylogenetic relatedness of the H5 HA gene between black bears and Eurasian-origin H5N1 HPAIVs, as well as representative subgroups of clade 2.3.4.4. (Appendix). The maximum clade credibility tree of the H5 HA gene showed that the HA genes of black bear samples were grouped with the current outbreak 2.3.4.4b subgroup (Figure 2). The HA genes were most closely related to those of early H5N1 HPAIVs isolated from eastern Canada provinces, as well as strains circulating in northwestern Europe during winter 2021. Those genes were also more similar to those the fully Eurasian lineages observed in red foxes than to those observed in striped skunks and mink (*5*) and were not closely related to the New England‒specific lineages documented in New England seals (*7*). Full genome sequences of both isolates were deposited in the GISAID database (https://www.gisaid.org) under accession nos. EPI_ISL_1747865 and EPI_ISL_17478584.

## Conclusions

The described black bears were within the Atlantic Americas avian flyway, where Eurasian lineage H5N1 viruses were detected in 2021 (*1*). The global epizootic of the HPAI H5N1 virus belonging to clade 2.3.4.4b has led to an exceptional number of animal deaths, particularly in domestic poultry and wild birds (*12*).

As opportunistic omnivores, black bears might be found scavenging on carcasses of dead animals, including birds. Within 5 km and in the 3 weeks preceding the euthanasia of both bears, several dead wild birds tested positive for the Eurasian lineage of HPAI H5N1, including common murre (*Uria aalge*), American crow (*Corvus brachyrhynchos*), northern gannet (*Morus bassanus*), and razorbill (*Alca torda*) (Canadian Wildlife Health Cooperative internal database, https://www.cwhc-rcsf.ca). Suspected deaths of seals caused by HPAI H5N1 had occurred around the same time that the bears were found; however, those seal carcasses were >300 km away, and no suspected or confirmed seal deaths caused by HPAI H5N1 have been reported in seal populations in the Gaspé Peninsula (*13*). Therefore, it is suspected that the adult female black bear in this study was infected through spillover directly from infected bird carcasses because black bears in the Gaspé Peninsula share habitat with marine birds for which there have been confirmed deaths caused by HPAI H5N1 (*13*; Canadian Wildlife Health Cooperative internal database) during the same period.

Although H5N1 virus transmission has been documented in mink and ferrets, transmission of the virus between mammals is generally inefficient (*14*). Therefore, the possibility that the virus that affected these black bears was transmitted from 1 bear to another, or from another mammal species, is much less probable than transmission from birds. Although HPAI virus infections in mammals might occur secondary to other infections, no other infectious agents were identified in either of the black bears.

Appendix Additional information on influenza A(H5N1) virus infections in 2 free-ranging black bears (*Ursus americanus*), Quebec, Canada.
