# Supplementary material for: Influenza A(H5N1) Virus Infections in 2 Free-Ranging Black Bears (Ursus americanus), Quebec, Canada
Source: Emerg Infect Dis. 2023 Oct;29(10):2145–9. doi: 10.3201/eid2910.230548 (PMC10521626; doi:10.3201/eid2910.230548)
Supplement: Appendix — Additional information on influenza A(H5N1) virus infections in 2 free-ranging black bears (Ursus americanus), Quebec, Canada. [file 23-0548-Techapp-s1.pdf]

Article DOI: https://doi.org/10.3201/eid2910.230548

EID cannot ensure accessibility for supplementary materials supplied by authors. Readers who have difficulty accessing supplementary content should contact the authors for assistance.

Influenza A(H5N1) Virus Infections in 2 Free-Ranging Black Bears (*Ursus americanus*), Quebec, Canada

Appendix

Materials and Methods

Phylogenetic Analysis

The H5 HA genes of two black bear samples were aligned with HA genes of Eurasian origin H5N1 HPAIVs as well as representative subgroups of clade 2.3.4.4 by using MUSCLE (*1*), and alignments were trimmed to contain coding regions. A Bayesian phylogenetic analysis was conducted to show phylogenetic relatedness by using a Bayesian Markov Chain Monte Carlo (BMCMC) method (*2*) as implemented in the BEAST 2 program version 2.7.4 (*3*). The GTR + G nucleotide substitution model was applied to the dataset for Bayesian analysis. The age of the viruses was defined as the date of sample collection from the dataset. The coalescent Bayesian skyline was used for tree prior and an uncorrelated log-normal relaxed clock model was used to reflect the complex population dynamics of AIVs. For the dataset, at least two independent BEAST analysis runs were conducted for 30 million generations, sampling every 3000 generations. Convergences and effective sample sizes (ESS) of the estimates were checked by using Tracer v1.7.2 (http://tree.bio.ed.ac.uk/software/tracer). All parameter estimates for each run showed ESS values >200. A maximum clade credibility (MCC) phylogenetic tree was generated to summarize all 10,000 trees after a 10% burn-in by using TreeAnnotator in BEAST 2 (*4*). The time-stamped phylogenetic tree was visualized and annotated by using FigTree v1.4.4 (http://tree.bio.ed.ac.uk/software/figtree).

Immunohistochemistry

For immunohistochemistry, paraffin tissue sections were quenched for 10 minutes in aqueous 3% hydrogen peroxide. Epitopes were then identified by using proteinase K for 15 minutes, then rinsed.

The primary antibody applied to the sections was a mouse monoclonal antibody specific for influenza A nucleoprotein (NP) (F26NP9, produced in-house) and was used at a dilution of 1:5,000 for 30 minutes. They were then visualized by using a horse radish peroxidase–labeled polymer, EnVision+ System (anti-mouse) (Dako, USA) and reacted with the chromogen diaminobenzidine (DAB). The sections were then counter stained with Gill's hematoxylin.

References

1. Edgar RC. MUSCLE: multiple sequence alignment with high accuracy and high throughput. Nucleic Acids Res. 2004;32:1792–7. PubMed https://doi.org/10.1093/nar/gkh340

2. Drummond AJ, Nicholls GK, Rodrigo AG, Solomon W. Estimating mutation parameters, population history and genealogy simultaneously from temporally spaced sequence data. Genetics. 2002;161:1307–20. PubMed https://doi.org/10.1093/genetics/161.3.1307

3. Bouckaert R, Vaughan TG, Barido-Sottani J, Duchêne S, Fourment M, Gavryushkina A, et al. BEAST 2.5: an advanced software platform for Bayesian evolutionary analysis. PLOS Comput Biol. 2019;15:e1006650. PubMed https://doi.org/10.1371/journal.pcbi.1006650

4. Drummond AJ, Suchard MA, Xie D, Rambaut A. Bayesian phylogenetics with BEAUti and the BEAST 1.7. Mol Biol Evol. 2012;29:1969–73. PubMed https://doi.org/10.1093/molbev/mss075

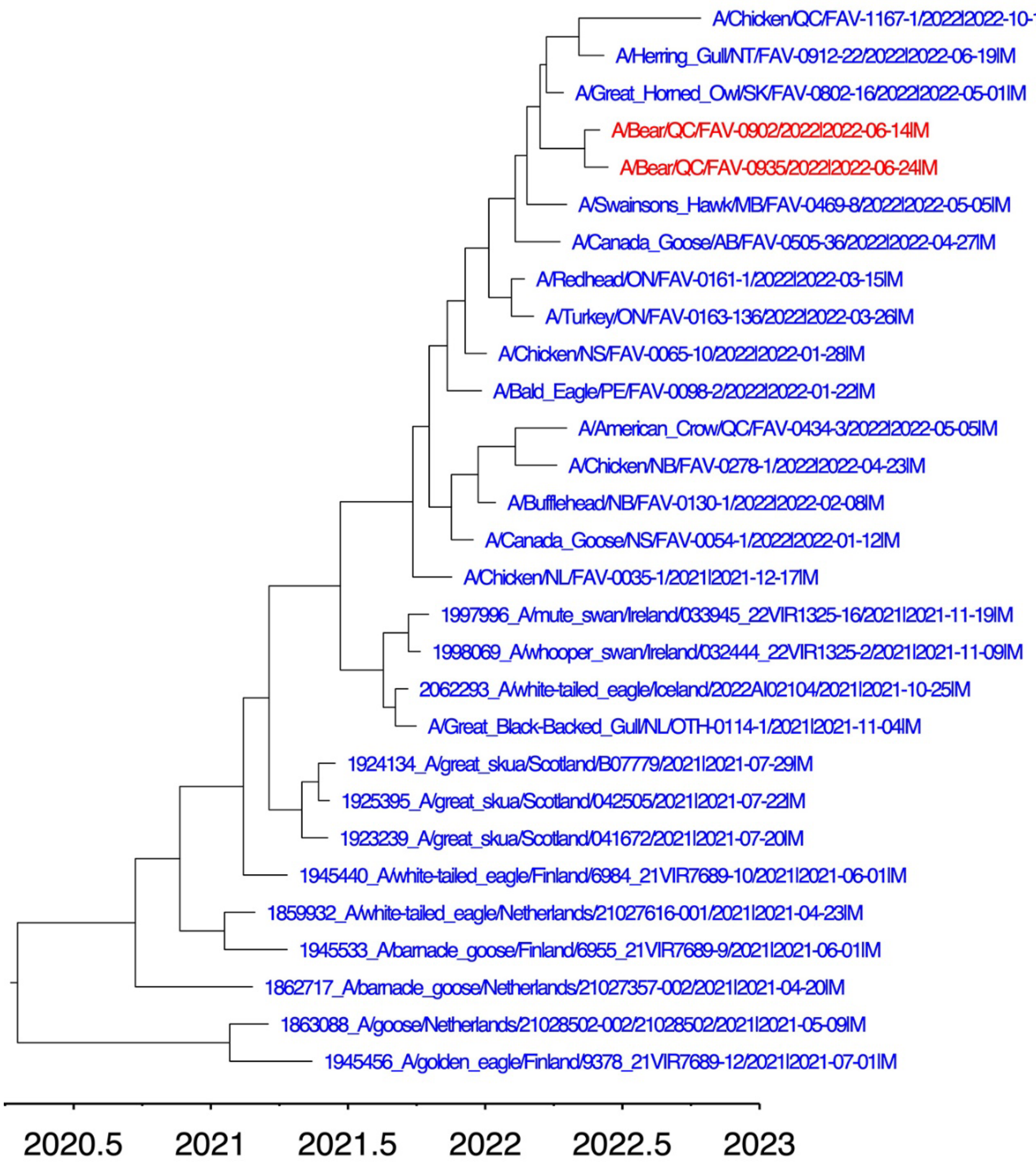

Appendix Figure 1. M (nt = 982) nucleotides from maximum clade credibility (MCC) tree inferred using Bayesian Markov Chain Monte Carlo (BMCMC) analysis for additional genomic segments from 29 strains described in 2.3.4.4b clade of Figure 2. Relationships among the European 2021 H5 2.3.4.4b HPAI strains (blue), the early Canadian wild birds and poultry strains (blue), and black bear strains (red).

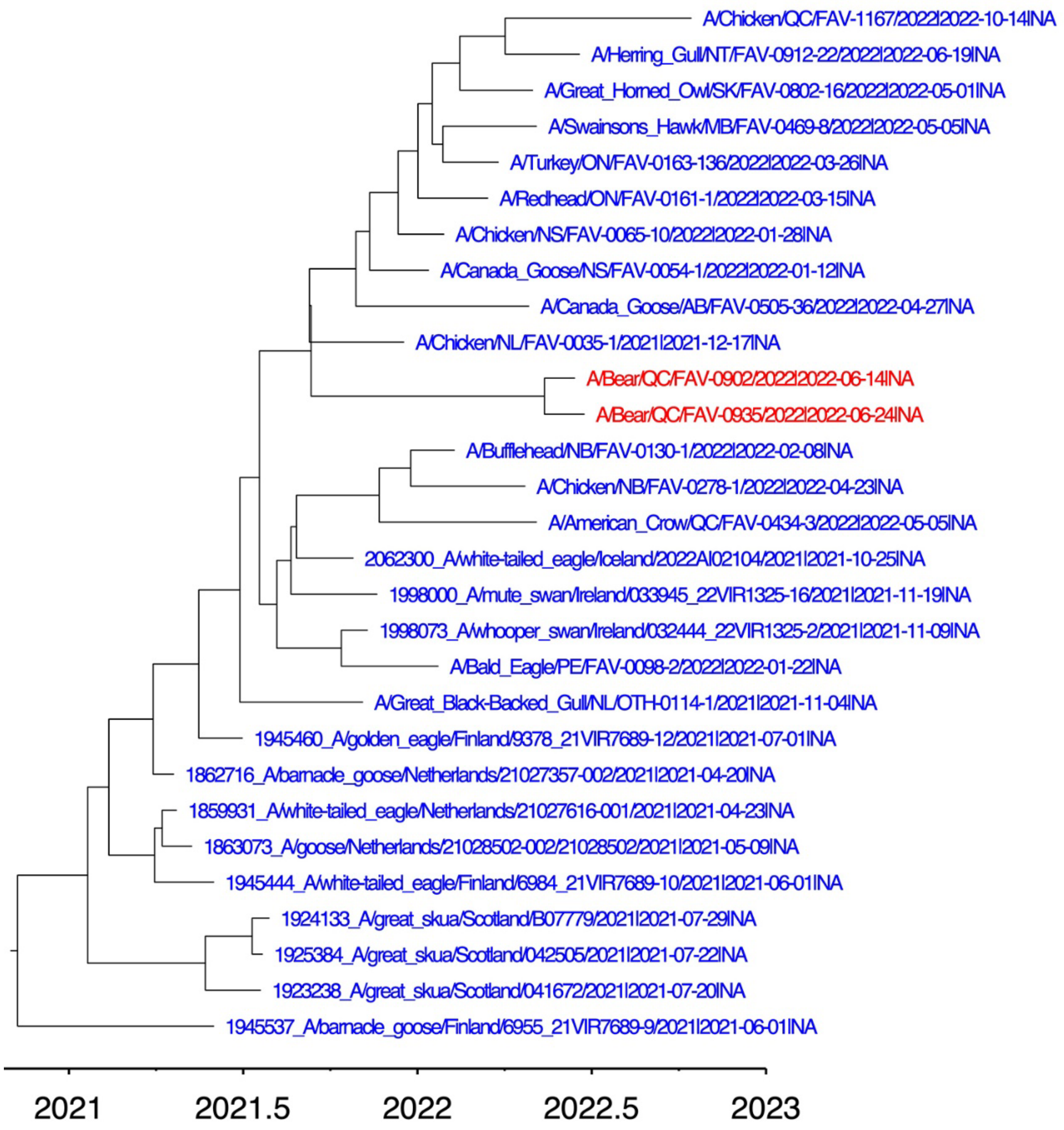

Appendix Figure 2. NA (nt = 1410) nucleotides from maximum clade credibility (MCC) tree inferred using Bayesian Markov Chain Monte Carlo (BMCMC) analysis for additional genomic segments from 29 strains described in 2.3.4.4b clade of Figure 2. Relationships among the European 2021 H5 2.3.4.4b HPAI strains (blue), the early Canadian wild birds and poultry strains (blue), and black bear strains (red).

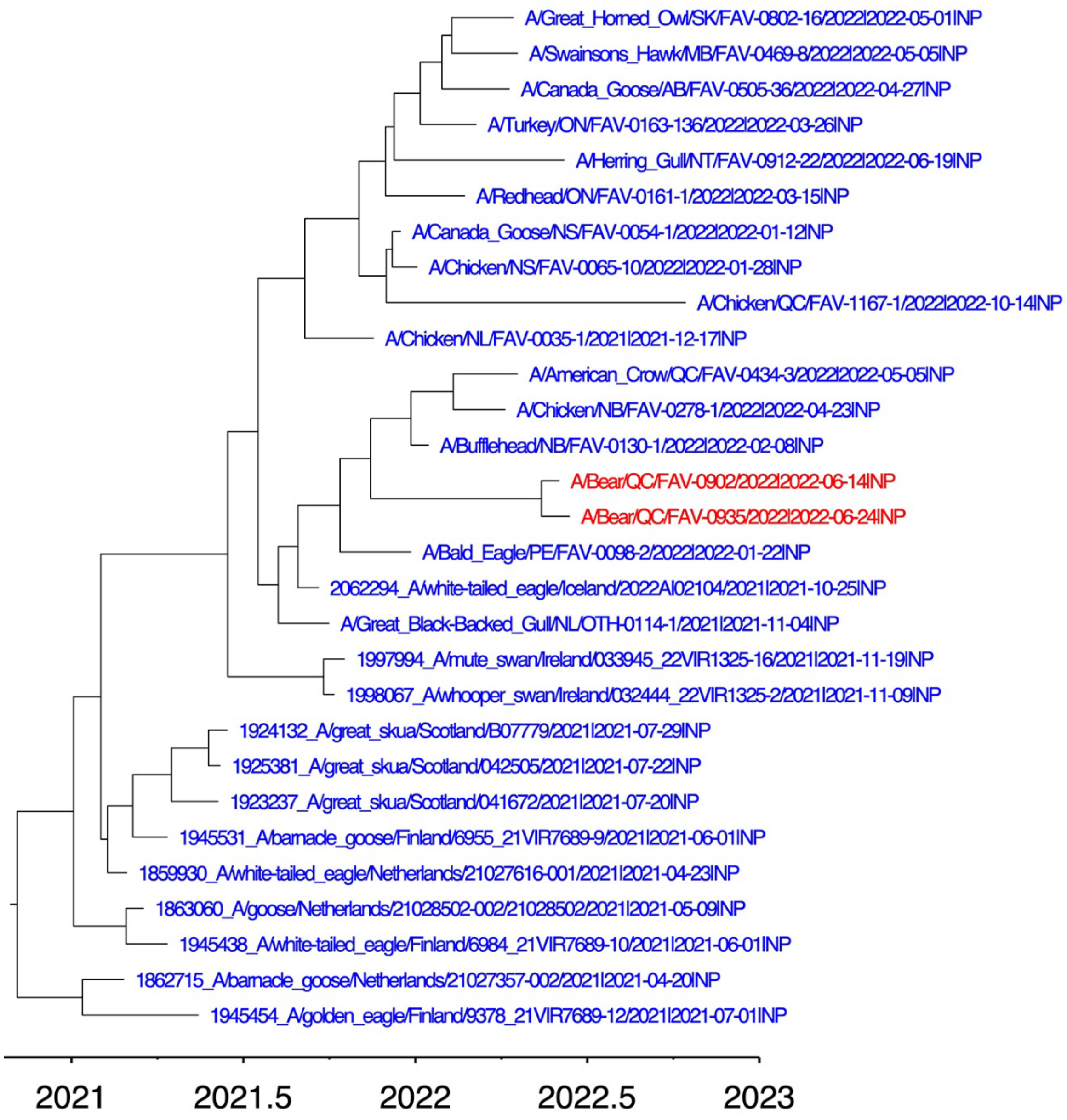

Appendix Figure 3. NP (nt = 1497) nucleotides from maximum clade credibility (MCC) tree inferred using Bayesian Markov Chain Monte Carlo (BMCMC) analysis for additional genomic segments from 29 strains described in 2.3.4.4b clade of Figure 2. Relationships among the European 2021 H5 2.3.4.4b HPAI strains (blue), the early Canadian wild birds and poultry strains (blue), and black bear strains (red).

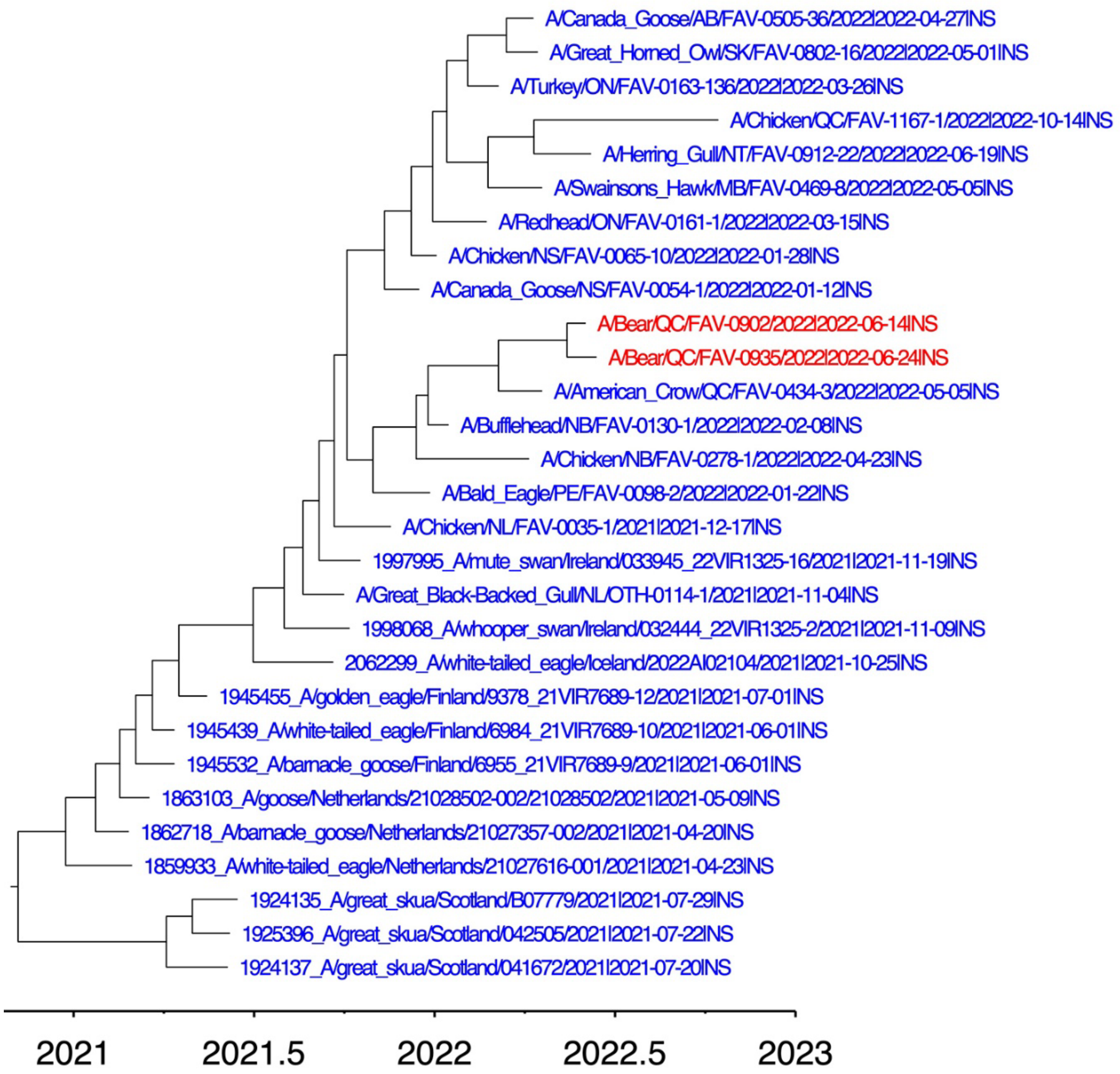

Appendix Figure 4. NS (nt = 838) nucleotides from maximum clade credibility (MCC) tree inferred using Bayesian Markov Chain Monte Carlo (BMCMC) analysis for additional genomic segments from 29 strains described in 2.3.4.4b clade of Figure 2. Relationships among the European 2021 H5 2.3.4.4b HPAI strains (blue), the early Canadian wild birds and poultry strains (blue), and black bear strains (red).

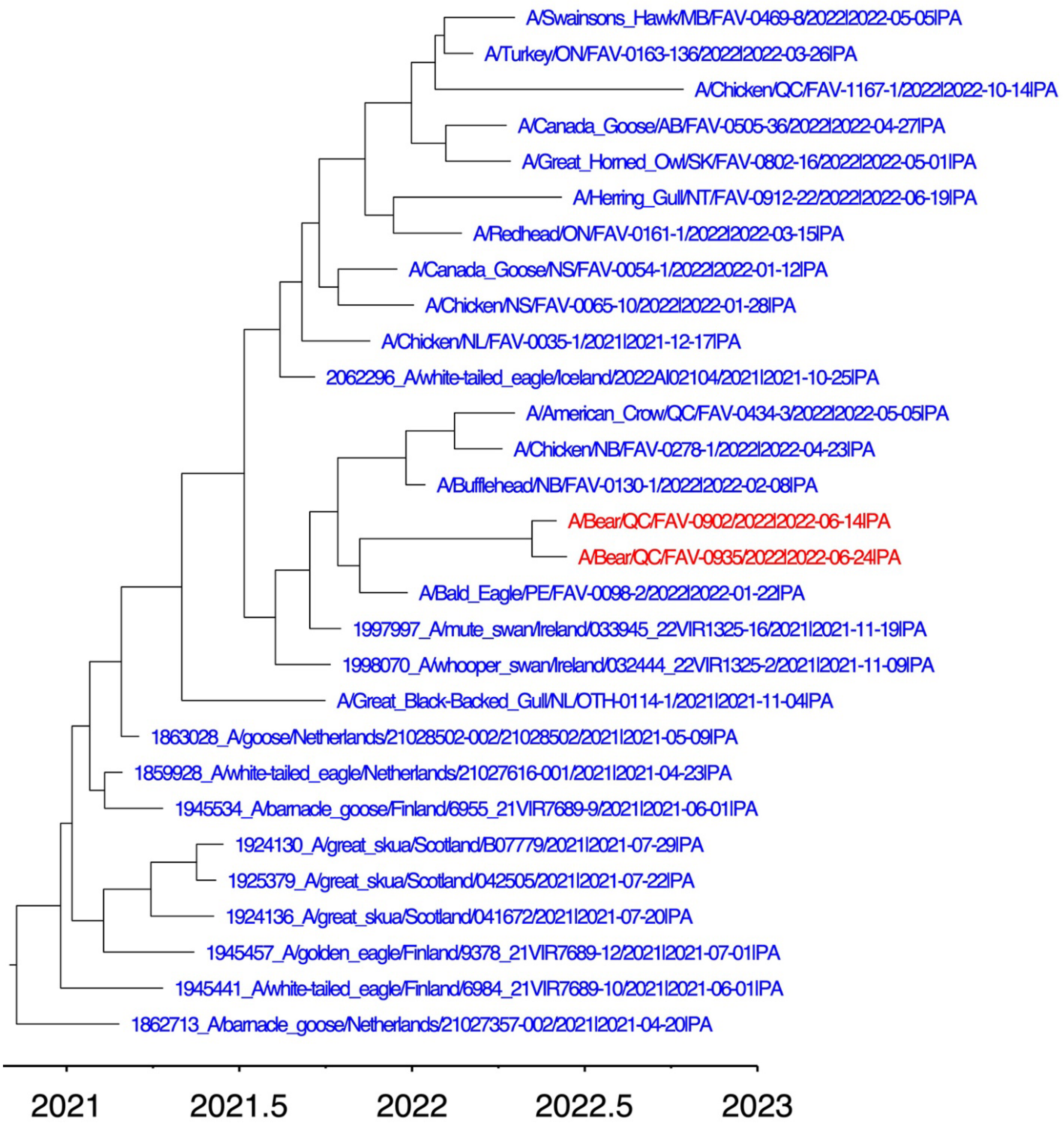

Appendix Figure 5. PA (nt = 2151) nucleotides from maximum clade credibility (MCC) tree inferred using Bayesian Markov Chain Monte Carlo (BMCMC) analysis for additional genomic segments from 29 strains described in 2.3.4.4b clade of Figure 2. Relationships among the European 2021 H5 2.3.4.4b HPAI strains (blue), the early Canadian wild birds and poultry strains (blue), and black bear strains (red).

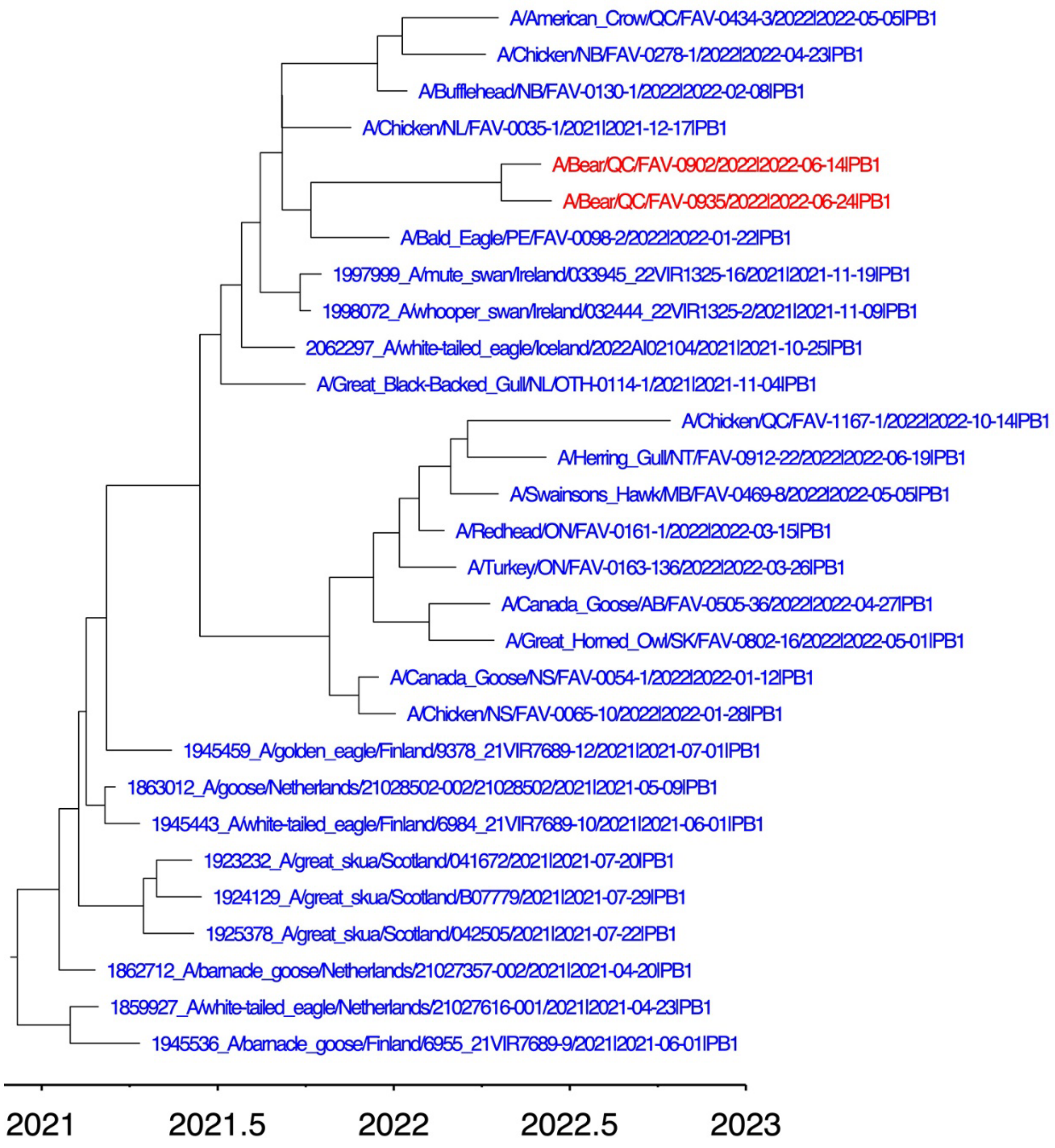

Appendix Figure 6. PB1 (nt = 2274) nucleotides from maximum clade credibility (MCC) tree inferred using Bayesian Markov Chain Monte Carlo (BMCMC) analysis for additional genomic segments from 29 strains described in 2.3.4.4b clade of Figure 2. Relationships among the European 2021 H5 2.3.4.4b HPAI strains (blue), the early Canadian wild birds and poultry strains (blue), and black bear strains (red).

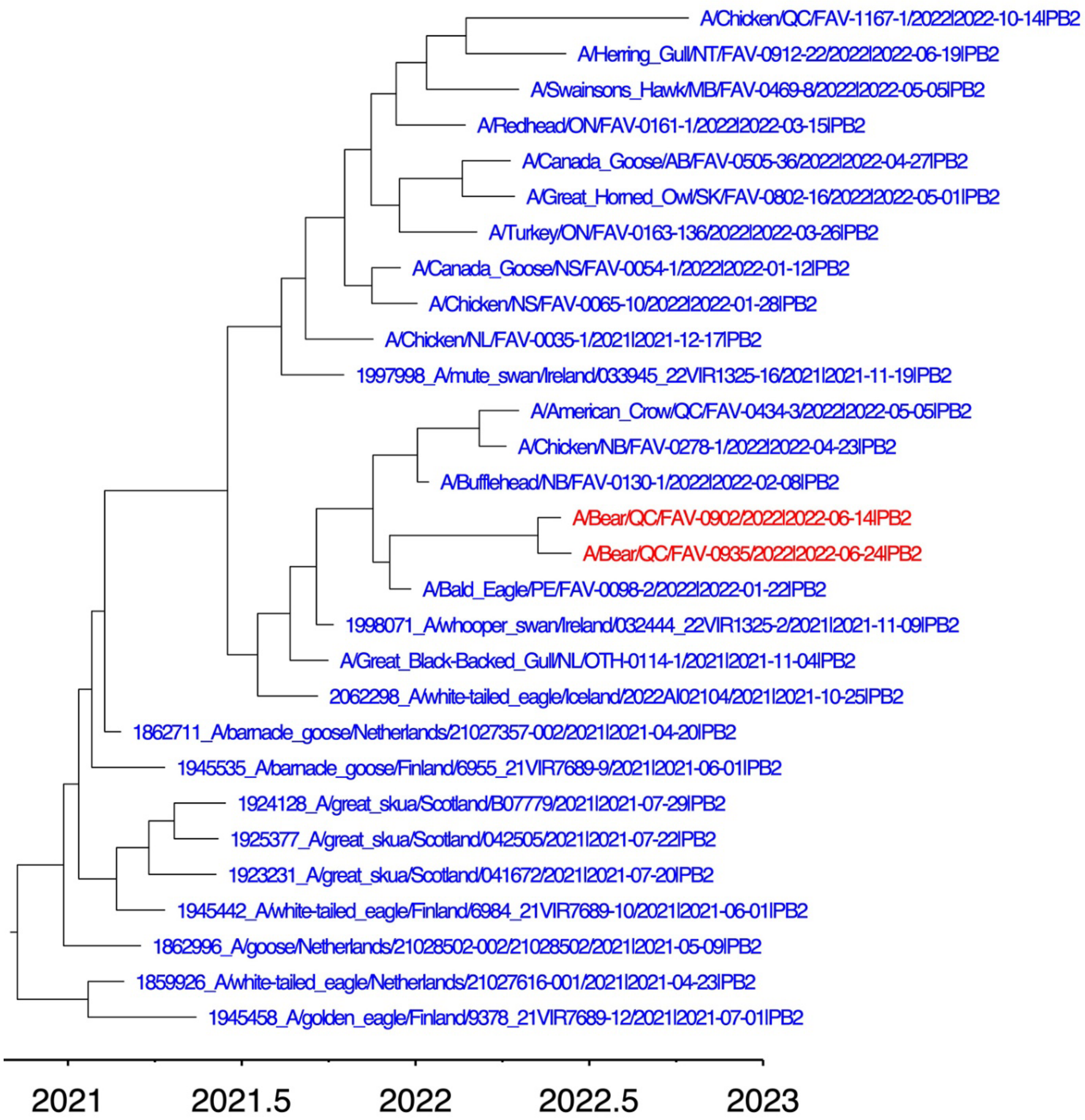

Appendix Figure 7. PB2 (nt = 2280) nucleotides from maximum clade credibility (MCC) tree inferred using Bayesian Markov Chain Monte Carlo (BMCMC) analysis for additional genomic segments from 29 strains described in 2.3.4.4b clade of Figure 2. Relationships among the European 2021 H5 2.3.4.4b HPAI strains (blue), the early Canadian wild birds and poultry strains (blue), and black bear strains (red).
